# First recourse for care-seeking and associated factors among rural populations in the eastern Democratic Republic of the Congo

**DOI:** 10.1186/s12889-021-11313-7

**Published:** 2021-07-10

**Authors:** Wyvine Ansima Bapolisi, Hermès Karemere, Freddy Ndogozi, Aimé Cikomola, Ghislain Kasongo, Albert Ntambwe, Ghislain Bisimwa

**Affiliations:** 1 Ecole Régionale de Santé Publique de Bukavu, Université Catholique de Bukavu, Bukavu, Democratic Republic of the Congo; 2 Division provincial de la Santé du Sud-Kivu, Bukavu, Democratic Republic of the Congo; 3 Programme RIPSEC (Renforcement Institutionnel des Institutions pour les Politiques de santé basées sur l’évidence en République Démocratique du Congo), Lubumbashi, Democratic Republic of the Congo; 4 Bureau central de la zone de santé de Walungu, Walungu, Democratic Republic of the Congo; 5 Ecole de santé Publique de l’Université de Lubumbashi, Lubumbashi, Democratic Republic of the Congo

**Keywords:** Care-seeking, Patterns of resort, Utilization, Health center, Traditional healer, The Democratic Republic of Congo

## Abstract

**Background:**

Access to quality healthcare is a global fundamental human right. However, in the Democratic Republic of the Congo, several parameters affect the choices of health service users in fragile, rural contexts (zones). The overarching aim of this study was to identify the first recourse of healthcare-seeking and the determinants of utilization of health centers (primary health care structures) in the rural health zones of Katana and Walungu.

**Methods:**

A cross-sectional survey was conducted from June to September 2017. Consenting respondents comprised 1751 adults. Continuous data were summarized using means (standard deviation) and medians (interquartile range). We used Pearson’s chi-square test and Fisher exact test to compare proportions. Logistic regression was run to assess socio-determinants of health center utilization.

**Results:**

The morbidity rate of the sample population for the previous month was 86.4% (*n* = 1501) of which 60% used health centers for their last morbid episode and 20% did not. 5.3% of the respondents patronized prayer rooms and 7.9% resorted to self-medication principally because the cost was low, or the services were fast. Being female (OR: 1.51; *p* = 0.005) and a higher level of education (OR: 1.79; *p* = 0.032) were determinants of the use of health centers in Walungu. Only the level of education was associated with the use of health centers in Katana (OR: 2.78; *p* = 0.045).

**Conclusion:**

Our findings suggest that health centers are the first recourse for the majority of the population during an illness. However, a significant percentage of patients are still using traditional healers or prayer rooms because the cost is low. Our results suggest that future interventions to encourage integrated health service use should target those with lower levels of education.

**Supplementary Information:**

The online version contains supplementary material available at 10.1186/s12889-021-11313-7.

## Background

Health care system efficacy is generally measured by how well prepared systems are to deal with calamities and epidemics under the best conditions [1]. About half of the world population does not have full coverage of primary health care services [2]. Some African countries, such as Angola [3], Ethiopia [3], Rwanda [4, 5], and Tanzania [6], have committed themselves to achieve the Sustainable Development Goals by offering Primary Health Care at a reasonable cost by subsidizing health facilities. These countries improved healthcare access and, as a result, they reduced child and maternal mortality by at least 50% [3, 6, 7].

Several studies, however, have found that other African countries, including the Democratic Republic of Congo (DRC), continue to struggle to provide high-quality care to their citizens [8–10].

Studies conducted in Ethiopia, Mali, Nigeria, and Uganda demonstrated that a variety of factors determine individuals’ patterns of resort of care when deciding to seek health care from health facilities or alternative (non-biomedical) providers [11–18]. These factors include age, occupation or income, gender, level of education, accessing transport, and high cost [11–15]. A number of these factors such as gender, level of education, cost, or income emerged consistently across studies.

Some studies showed that the presence of alternative medicine, such as informal health structures, prayer chambers, and/or traditional healers, were considered less invasive and less expensive than biomedical health facilities, which has constituted a barrier to the use of integrated health facilities [15, 17, 18]. Some populations, mostly poor and rural, gravitate towards alternative medicine rather than health facilities [19–21]. Traditional healers or non-integrated health facility services may be less expensive but, with little to no state regulation, they can also be harmful to the population’s health [3, 22].

In the DRC, the national, provincial, and local health systems face ongoing challenges. The health sector in DRC is essentially funded from three sources: the State (about 3% of the National health budget), user fees (up to 70% of the health budget), and external (bilateral and multilateral) contributions [23, 24]. Health sector regulation can be difficult given the increasing number of privates, mainly religious organizations managing health facilities and, in some cases, entire health zone systems. In the face of these challenges, the DRC government is making tangible efforts to rebuild the health system and coordinate all of the actors implicated in health system improvements [24, 25].

Six main principles drive the current national strategy to increase population coverage of primary health care: (1) revitalization of the health zone and correction of distortions at peripheral level; (2) reorganization of the central and intermediate levels of the health system; (3) rationalization of health financing; (4) strengthening intra- and inter-sectoral partnerships; (5) development of human resources for health; and (6) improving research in health systems [24].

However, despite ongoing governmental and non-governmental organizations’ (NGO) efforts to improve health system quality and coverage, the utilization of integrated health facilities remains low, around 33% [19, 26]. The population is impoverished and encounters barriers in accessing care including the cost of user fees [22, 27, 28]. Ongoing political instability and civil conflicts, mainly in the eastern part of the country, worsen the situation.

In order to build a responsive health care system, there is a need of understanding health-seeking behavior at a population level. In this study, we focused on socio-determinants of health care utilization in two rural health zones in South-Kivu, eastern DRC. This study aims to investigate the first recourse of care and the socio-determinants of the utilization of health centers (primary healthcare facilities) in poor, rural, and post-conflict zones. This analysis can contribute to the planning and implementation of interventions for health systems strengthening and services use in rural regions.

## Methods

### Study design

This study is a comparative cross-sectional study. We conducted a survey over 3 months, from June to September 2017.

### Study setting

**Fig. 1:**
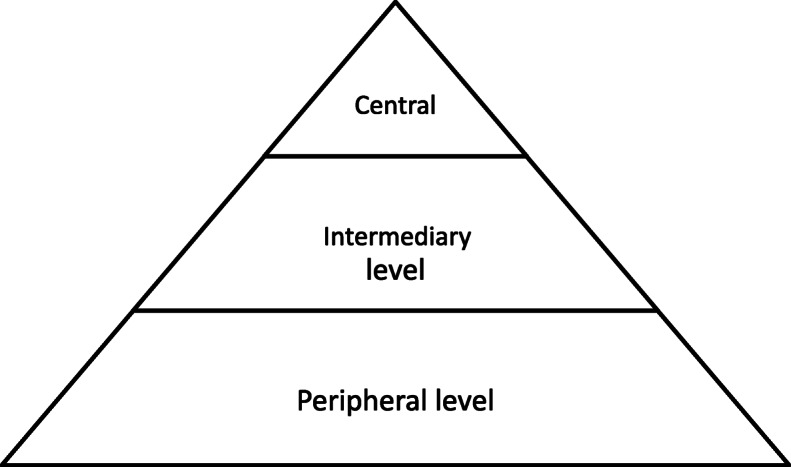
Health system organization in the DRC. 1. Central (strategic level): (Ministry of Health comprising the Minister, the General Secretary, Central Directorates, and specialized services. 2. Intermediary: the mid-point or bridge (ensures the implementation of national policies and identifies and promotes the needs of different health zones). 3. Peripheral (health zones). The health zones are the operational level of providing healthcare via health centers

The DRC health system is currently organized as a pyramidal structure with three levels [25, 29, 30] (Fig. 1).

**Fig. 2:**
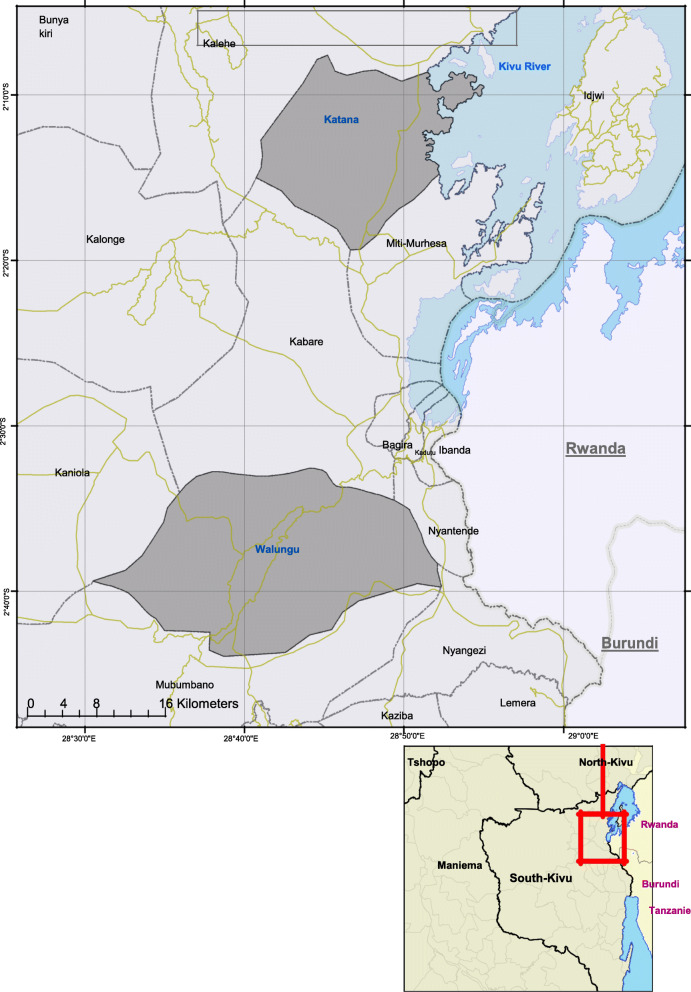
Katana and Walungu health zones in South Kivu, DRC

The ‘health zone’ in DRC corresponds to a ‘health district’ in other contexts.

The three levels of the health system in DRC [25, 29, 30].

### Relevant definitions

Table 1 contains definitions of some concepts used through the manuscript.

**Table 1:** Definitions of some concepts [30]

**Health zone**	A well-delimited entity (maximum diameter 150 km) contained within the limits of a territory or an administrative commune with a population of at least 100,000 inhabitants (with similar socio-cultural characteristics). A health zone provides health services at two interdependent levels (health centers at the first echelon and a General Reference Hospital at the 2nd echelon), under the supervision of a health zone Management Team.
**Health Centre (primary health care structure)**	A peripheral public health care structure that is run by at least one nurse (enrolled nurse or midwife, secondary education graduate). Its mission is to provide good quality integrated health care to a population (generally 10,000 inhabitants) within a defined geographical health area (less than 5 km by airway). The health center serves as the first contact with health systems offering a minimum package of care (consultation, basic curative care, maternity, immunization activities, and laboratory). Patients or vulnerable people are referred to the General hospital of Reference for special care such as surgical interventions to ensure that a better care pathway is followed (complementary package of care).
**Health area**	A geographic entity of the delimited health zone, composed of a set of villages in rural areas and/or streets in urban areas, established according to socio-demographic affinity criteria, with an average population size of 10,000 inhabitants depending on the area (rural or urban). A health center covers one health area
**Reference health center**	Any health care structure that carries out some specific activities usually provided by the General Hospital of Reference in addition to traditional health centers’ attributions. A reference Health Center is managed by a Medical doctor or at least a qualified nurse (with an advanced diploma or a degree).

The study took place in the rural health zones of Walungu and Katana (Fig. 2)

The Walungu health zone covers an area of 800 km^2^ with 254 villages. The population was estimated at 294,527 inhabitants in 2017 [31].

Walungu has 23 health areas with 12 confessionals, three reference health centers, and one general reference hospital. The health zone receives insufficient funding from user fees and state subsidies while external financing is almost non-existent [32].

The health zone of Katana covers an area of 400 km^2,^ with 118 villages. In 2017, the population was estimated at 244,332 inhabitants [31]. Katana has 18 health areas with nine confessionals, three reference health centers, a hospital center, and a reference hospital. The health zone depends almost entirely on user fees and external funding from international NGOs, although this aid is in decline [33].

The inclusion of the two health zones as pilot sites for the upcoming reforms by the *Renforcement Institutionnel des Institutions pour les Politiques de santé basées sur l’évidence en République Démocratique du Congo* (RIPSEC) and accessibility drove our decision to include these zones in this study. The relative difference in financing of the health zones also motivated our choice: compared to Walungu, Katana has more funding as a result of long-lasting partnerships with international NGOs.

### Study population

#### Inclusion criteria

The head of the family or his representative was recruited for the survey. In their absence, an individual > 15 years old regularly residing in the household was interviewed.

#### Sample size calculation

In a health zone with highly variable village population sizes, the World Health Organization’s (WHO) reference manual for designing cluster household surveys recommends using a 30-cluster sample (villages in our case) for service coverage in health at the national or regional level [34, 35]. Accordingly, we randomly selected 30 clusters between all the villages in each health zone. The sample frame was the list of villages for each health zone and related populations. All inhabitants fulfilling the inclusion criteria in the selected clusters (villages) had the same chance to be included.

We used probability proportional to size (PPS) for sampling, which means that villages with larger populations were likely to be recruited more than one time. PPS was used in similar settings when the size of villages was highly variable [28, 36].

The sample size was calculated using Schwartz’s formula [37]. WHO used this formula in a multi-country study assessing the utilization of health facilities in Africa, including DRC [8, 38]. We calculated the sample size for each site using this formula:

$$ \mathrm{n}={\mathrm{z}}^2\ast \mathrm{p}\ast \mathrm{q}\ast \mathrm{e}/{\mathrm{d}}^2 $$

Where:

n: Minimum sample size to obtain significant results for an event and a fixed level of risk.

z: Confidence level (the standard value of the 95% confidence level is 1.96).

p: Probability of occurrence of the event or prevalence was assumed at 50% for attendance of health centers as there is no prevalence of the attendance of health facilities available for the region.

d: Margin of error (usually set at 5%).

e: Sample design effect (cluster effect = 2.3).

The sample size calculated was 884 households per health zone. Assuming attrition of 5%, we extended the sample size to 928 households per health zone.

The number of people to be interviewed per village was obtained by dividing the sample size by the number of clusters (30) equaling 31 households (one person per household) to be surveyed per village. A central location was chosen at random and designated in each of the villages to serve as a starting point for data collection. From the starting point, research assistants turned right at each crossroad until they reached the desired number of respondents. If the required number was not reached, the investigators went to a neighboring community to complete the sample. Ultimately, 1751 consenting people (viz.*,* 961 from Katana; 790 from Walungu) participated in the study.

In Katana, we achieved the estimated sample size. Walungu, however, yielded a response rate of 89.4%. This difference can be explained by difficulties reaching some households in Walungu, agricultural activities that took potential participants far from their homes during survey completion, and participant reticence due to regional insecurity. Walungu is among regions of the South-Kivu province characterized by ongoing conflicts with recurrent attacks in the civil community. Researchers were also encouraged to leave the field earlier in Walungu due to security concerns.

#### Data collection

Data were collected using a semi-structured interviewer-administrated questionnaire at the household level. Seven research assistants were trained in data collection methods and participated in a pilot test before the start of data collection.

#### Instruments

The questionnaire (Additional file 1) was developed based on previous studies conducted in poor settings [13, 14, 39]. Content validity was assessed by public health experts from the *Ecole Régionale de Santé Publique de Bukavu* (Content validity index = 0.96). Test-retest reliability of the questionnaire was obtained by administering the questionnaire two times to the same 30 households after 2 weeks interval in a rural health zone, Miti-Murhesa. The survey collected information on participant socio-demographics, motivations for choosing a particular structure for care in case of illness, and the use of healthcare services within the community.

An ecological square of medical care adapted from White to determine the percentage of the population that accessed a health center (nurse) and hospital (physician) was drawn [40]. The purpose of White’s square model is to advocate for the training of more physicians so that every person with a health need has access to a physician [40].

#### Measures

The dependent variable was the utilization of health centers. It was assessed by a yes-no question, “*during the last 30 days, did you use the health center seeking care?*”

The explanatory variables were age, sex, married or not, educational level, and employment status (Table 2). The details on measurements of all variables in the study are provided in Table 2 and the questionnaire (Additional file 1).

**Table 2:** List of variables

Variable	Definition	Type of variable	Units
**Primary outcomes**			
Utilization of health center (primary outcome)	during the last 30 days, did you go to the health center seeking care?	Binary	Binary (yes/no)
First resort during last illness episode (primary outcome)	During your last illness episode, where did you go seeking care?	Nominal	Self-medication, traditional healer (provider of care who uses plants and traditional rites to cure disease), prayer room, Health center, private, none.
**Explanatory variables**			
Sex	Sex of respondent	Binary	Female = 1/Male =0
Age	Age of respondent	Quantitative	Years
Health Zone	Heath zone where the respondent resides	Nominal	Katana, Walungu
Marital status	Matrimonial statute	Nominal	Single, married, widow (er), divorced Coded married =1, married =0 otherwise
Profession	Occupation or activity	Nominal	None, cultivator, Teacher, state employee
Education level	Last level reached by the respondent	Ordinal	None = 0 Primary = 1 Secondary = 2 Higher education (tertiary) =3
During the last 30 days, were you ill?	Were you ill in the last 30 days	Binary	Yes =1, No = 0
Motivation for the choice of health care structures	Main reason for choosing a particular structure	Nominal (multiple choice)	close to my home; cheaper/less expensive; financial advantage; the service is fast, I was looking for a specific receiver; Recommended by a third party. Other please precise … feel more confident (if hospital), Modern medicine is not able to cure this disease (traditional healer, prayer room)
Combination of another type of medicine for health center users	Did you use any other structure or type of medicine prior coming to the health center?	Nominal	Prayer room, private structures, or traditional healer before going to the health center

The first recourse for care was defined as the first place the person went to seek care in case of illness (health center, hospital, traditional healer, prayer room, private or nowhere); a private health facility was defined as a health facility belonging to individual providers *not* integrated into the health system.

We asked participants who chose the health center which services they accessed, and if they also consulted with traditional healers, a prayer room, or a private provider. Participants who had a consultation in a structure other than a health center were asked to give their motivation for seeking care outside of the health system (multiple-choice question with an option for ‘Other’ and a specified explanation).

#### Data analysis

Stata version 14/Statacorp software was used for analysis. Data were summarized using means (standard deviation) and medians (interquartile range) for continuous data with 95% confidence intervals. We summarized categorical variables in terms of frequencies with their proportions. We used Pearson’s chi-square test to compare proportions for categorical variables; for a variable with less than 5 in a cell, we used Fisher exact test. The comparison of means was performed using Student’s t-test. The alpha level was set at *p* < 0.05.

We used logistic regression to determine the relationship between socio-demographic factors and the use of health centers (outcome variable). The goodness-of-fit was assessed using Hosmer-Lemeshow (Hosmer-Lemeshow Chi^2^ = 8.3, *p*-value = 0.40). We retained the model with the lower Akaike’s information criterion (AIC). Observations with missing data for the variables of interest were discarded. The Association with a *p*-value less than 0.05 was deemed significant.

#### Ethical considerations

Ethical approval was obtained from the Catholic University of Bukavu’s Ethics Committee. Before the beginning of each survey, the study was fully explained and each interviewee gave his/her written informed consent to participate. Additionally, for participants under the age of 18 years, written consent was obtained from a parent or legal tutor. Respondents were guaranteed confidentiality, as well as the absence of risk related to participating in the study.

## Results

### Socio-demographic characteristics of the study population

Women comprised the majority of respondents (53.7%, *n* = 941). The age of participants ranged from 15 to 77 years. The population of Walungu, with a median age of 27 years (IQR 23–37), was younger than that of Katana, with a median age of 30 years (IQR 25–40, *p* = 0.001). 59.6% (*n* = 1043) of respondents were married. 39.0% (*n* = 680) of the study population reported being unemployed and 39.1% (*n* = 683) were farmers (Table 3).

**Table 3:** Socio-demographic characteristics of the respondents

Variables	Katana (%) (***n*** = 961)	Walungu (%) (***n*** = 790)	***χ***^**2**^	p-value	Total (%) (***n*** = 1751)
**Age median (range)**	**30(25–40) years**	**27(23–37) years**		**0.001**	0
**Sex**					
Female	511(53)	430(54.4)	0.27	0.600	941(53.7)
Male	450(47)	360(45.6)			810(46.3)
**Marital status**	***n*** **= 961**	***n*** **= 790**			***n*** **= 1751**
Single	281(29.2)	285(36.1)	64.21	**< 0.001**	566(32.3)
Divorced	5(0.5)	24(3.0)			29(1.6)
Married	639(62.5)	404(51.1)			1043(59.6)
Widow (er)	36(3.7)	77(9.8)			113(6.5)
**Profession**	***n*** **= 961**	***n*** **= 784**			***n*** **= 1745**
State employee	217(22.6)	165(21.0)	34.20	**< 0.001**	382(21.9)
Cultivators	426(44.3)	257(32.8)			683(39.1)
Unemployed	318(33.1)	362(46.2)			680(39.0)
**Education level**	***n*** **= 961**	***n*** **= 788**			***n*** **= 1749**
None	312(32.5)	203(25.8)	48.60	**< 0.001**	515(29.5)
Primary	238(24.8)	210(26.6)			448(25.6)
Secondary	378(39.3)	284(36.1)			662(37.8)
Higher education	33(3.4)	91(11.5)			124(7.1)

### The first recourse of seeking care and driving motivation of the choice

**Fig. 3:**
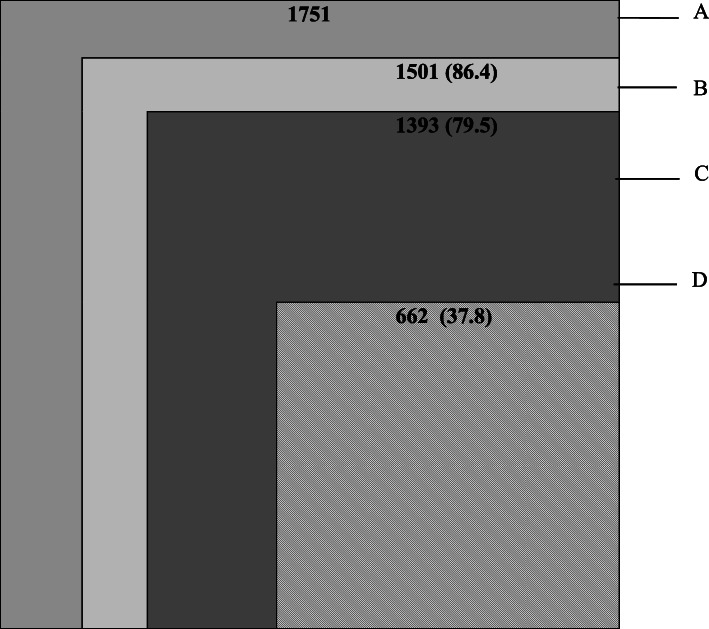
An adaptation of White’s square model for utilization of health structures. **A** Population at risk. **B** People who reported a health problem. **C** People consulting a hospital or a health center. **D** Patients hospitalized in health centers

The study revealed that 1501 people (86.4% of the study sample) felt sick during the last month (Fig. 3). Referring to the White’s square model, 79.5% (*n* = 1393) of the study population used integrated health facilities (health centers or hospitals) during the last episode of illness (Fig. 3).

Nearly 60% (*n* = 1041 patients) of those who resorted to healthcare facilities used health centers, and 20% (*n* = 352) used hospitals. 24% (*n* = 186) of the total study population in Walungu (vs. 17% (*n* = 166) in Katana, *p* = 0.002) reported preferring hospitals as the first point of care (Table 4). Reasons for the patronage of hospitals included having confidence in a big hospital (66.4%, *n* = 234) and closer proximity to the hospital than to a health center (18.2%, *n* = 64).

**Table 4:** First recourse of care

Variables	Katana (%) (***n*** = 961)	Walungu (%) (***n*** = 790)	***χ***^**2**^	P	Total ***n*** = 1751
Sick during the last 30 days	856(89.1)	645(85.8)			1501(86.4)
**Place of first resort for care**	***n*** **= 961**	***n*** **= 776**			***n*** **= 1737**
Self-medication	77(8.0)	61(7.9)	21.44^a^	0.002	138(7.9)
Prayer room	40(4.2)	39(5.0)			79 (4.5)
Health center	618(64.3)	423(54.5)			1041(60.0)
Hospital	166(17.3)	186(24.0)			352(20.3)
I did nothing	4(0.4)	7(0.9)			11(0.6)
Private (dispensaries, etc.)	13(1.4)	11(1.4)			24(1.4)
Traditional healers	43(4.5)	49(6.3)			92(5.3)

^a^ Fisher exact test

The proportion of utilization of self-medication, private facilities, prayer rooms and traditional healers comprised 8% (*n* = 138), 1.4% (*n* = 24), 4.5% (*n* = 79) and 5.3% (*n* = 92) respectively.

Reasons for recourse to structures other than the health center are presented in Table 5. Prayer rooms and/or traditional practitioners were preferred because they were faster in providing care, or a third party recommended them (Table 5).

**Table 5:** Preference of types of care during the last illness episode

Reason for choosing	Katana (%)	Walungu (%)	***χ***^**2**^	P	Total
**The hospital**	**(*n*** **= 166)**	**(*n*** **= 186)**			**(*n*** **= 352)**
Financial advantage	18(10.8)	14(7.5)	13.12^a^	0.022	32 (9.1)
Specific receiver	4(2.4)	18(9.7)			22(6.3)
I am more confident	107(64.5)	127(68.3)			234(66.4)
Closer to my home	37(22.3)	27(14.5)			64(18.2)
**Private**	***n*** **= 10**	***n*** **= 13**			***n*** **= 23**
Financial advantage	4(30.8)	4(57.1)	1.31^a^	0.250	8(40.0)
Less expensive	9(69.2)	3(42.9)			12(60.0)
**Prayer room**	***n*** **= 40**	***n*** **= 37**			***n*** **= 78**
Modern medicine not able to heal	7(18.9)	6(22.2)	10.57	0.005	13(20.3)
Recommended by a third party	9(24.3)	16(59.3)			25(39.1)
Rapid service	21(56.8)	5(18.5)			26(40.5)
**Self-medication**	***n*** **= 77**	***n*** **= 60**			***n*** **= 137**
Less expensive	37(48.0)	24(46.2)	0.04	0.970	61(47.2)
Easier	20 (26.0)	14(26.9)			34(26.4)
Everyone is doing it	20 (26.0)	14(26.9)			34(26.4)

^a^ Fisher exact test

Patients who used private facilities were motivated by the low cost of care.

### Utilization of health centers

38% (*n* = 236) of the patients in Walungu and 33.5% (*n* = 145) of the patients in Katana who used health centers did at least twice a month before the survey. In Walungu, 4.5% (*n* = 18) of sick people who consulted a health center admitted to having used traditional healers/prayer rooms or private before going to the health center, while in Katana, that rose to 10.9% (*n* = 49) of patients. The reasons for visiting health centers in both health zones were general consultations followed by antenatal care (ANC) (Table 6).

**Table 6:** Health centers and service attendance during last illness episode

Variables	Katana ***n*** = 961	Walungu ***n*** = 790	***χ***^**2**^	P
Use traditional healers/prayer room before going to health center	49(10.9)	18(4.5)	10.14	0.001
Attendance of health center	618(64.3)	437(55.4)	8.12	< 0.001
Use the health center at least twice	236 (38.2)	145 (33.1)		
Stay at least one night in health center	408(66.0)	254(55.9)	6.36	0.010
**Services utilized**	***n*** **= 618**	***n*** **= 438**		
Consultation	469(75.9)	304(69.4)	10.93	0.004
ANC	120(19.4)	92(21.0)		
Other	29(4.7)	42(9.6)		
Women who had given birth recently	334(54.0)	263(58.0)	1.70	0.192

Health centers in Katana were used more frequently compared to those in Walungu, 64.3% (*n* = 618) and 55.4% (*n* = 437) respectively, *p* < 0.001 (Table 6).

More than half (54%, *n* = 334 in Katana and 58%, *n* = 263 in Walungu, *p* = 0.192) of women from participating households recently gave birth at the health centers.

### Determinants of the utilization of health centers

Table 7 shows the results of multiple logistic regression analyzing determinants of health center use. Being a female (adjusted OR = 1.51, *p* = 0.005) and having a tertiary level of education (adjusted OR = 1.79, *p* = 0.032), had independent effects on the probability of using health centers in Walungu. Only tertiary level education (adjusted OR = 2.78 and *p* = 0.045) in Katana showed an independent effect on the utilization of health centers.

**Table 7:** Multiple logistic regression of determinants of the utilization of health centers

Variables	Katana				Walungu			
	Odds ratio	P	95% CI		Odds ratio	p	95% CI	
Age (years)	0.99	0.982	0.98	1.01	1.00	0.348	0.99	1.01
Sex	1.03	0.786	0.79	1.36	**1.51**	**0.005**	1.13	2.03
Married	1.29	0.101	0.95	1.75	1.28	0.104	0.94	1.74
Employment								
None	Reference					Reference		
Farmer	0.75	0.093	0.53	1.04	1.00	0.980	0.69	1.44
State employee	0.98	0.960	0.66	1.47	0.83	0.380	0.56	1.24
Education level								
None	Reference					Reference		
Primary	0.95	0.806	0.66	1.37	1.07	0.726	0.72	1.59
Secondary	0.80	0.216	0.56	1.13	1.08	0.673	0.74	1.58
Tertiary	**2.78**	**0.045**	1.02	7.58	**1.79**	**0.032**	1.05	3.05

## Discussion

The objective of this study was to identify the first recourse of care and the determinants of utilization of health centers (primary health care structures).

### Choice of first recourse and motivation

The percentage of subjective morbidity using White’s square (86.4%) is a bit higher than what White found in 1961 (75%) but quite similar to the findings of Green et al. and Thacker et al. (80 and 86% respectively) [41, 42]. The high prevalence of subjective morbidity suggests that the population is facing numerous health problems. The percentage (80%) of respondents, which consulted an official health structure (health center or hospital), is similar to that found in the literature on other sub-Saharan African countries [14, 43]. The slight differences between the two health zones included in the study (Katana: health centers 64.3% and hospital 17.8% vs Walungu health centers 54.5% and Hospital 24.0%) could be explained by the relative variation in financial support between the two health zones, with Katana being better funded than Walungu. However, other considerations, such as ease of physical access to health centers, could also influence user choice. Walungu hospital’s central location could explain why it is more patronized compared to Katana hospital, which is not centrally located and more difficult to access than many health centers.

The percentage of respondents (80%) who consulted an official health structure in this study is higher than findings from Kinshasa (33%), the capital of DRC, and the Demographic and Health Surveys [19, 26]. One of the reasons that could explain a high percentage in South Kivu, compared to the other regions in the country, is the management of the health areas by *Bureau Diocésain des Oeuvres Médicales* (BDOM). BDOM is a Catholic NGO providing financial support for the state for several decades. This NGO, with the support of many partners, subsidizes health centers, which allow them to offer services at a lower price. In many health facilities around the country, patients must pay before receiving care and pay a fee for each service rendered. Patients at health facilities managed by BDOM, however, do not have to pay before receiving care and pay a flat fee for all services.

For participants who sought health services from private structures, prayer rooms, or traditional healers, the primary reason was the lower cost. Our results are similar to those found in the literature; the high cost of care was described in many studies as the first reason to under-utilize health facilities [22, 27, 28, 43]. In previous studies in the DRC, an increase in service uptake was observed after the lowering of user fees [22, 27, 44]. Unlike what was found in other Sub-Saharan countries [45, 46], few respondents in this study reported combining the use of two different structures (integrated health structures or traditional or prayer rooms). Pervasive poverty may also explain why individuals cannot afford to pay users fees at two types of health facilities at the same time. They only go to official health facilities (more expensive) when absolutely necessary.

In both health zones awareness campaigns educating the population on the harmful effects of “quacks”, or individuals posing as alternative or biomedical healers without licensed medical training, are prevalent. The low percentage of those in our study who consulted private providers, traditional healers, or prayer rooms could be a result of these campaigns [47]. Similarly in Uganda, the use of traditional healers decreased after mass education campaigns against unlicensed healers [48].

Social desirability bias, namely feeling ashamed to reveal the use of several types of care, can also be an explanation for the low percentage of participants consulting alternative providers. Especially in the context of campaigns against ‘quacks’, participants could be reluctant to admit that they sought care from unrecognized health structures.

### Socio-determinants of the utilization of health centers

Logistic regression showed that in Walungu the level of education (higher level) and being female were independent determinants of the utilization of health centers. In Katana, only the level of education (higher level) had an independent effect on the attendance of health centers.

These results are similar to those found by other researchers. Many authors have described the level of education as a key determinant of the utilization of primary healthcare services [13, 49, 50]. Higher-level educated people may possess higher levels of self-efficacy and therefore be more equipped to make informed choices when seeking care [51, 52]. Being female was also found to be a determinant factor for utilizing health centers (primary healthcare), which is mainly explained by childbearing age [14]. The fact that ANC was the second most utilized service at health centers, as found in our results, reinforced this hypothesis. Women are more likely to use health centers when seeking gynecological or obstetrical services. South-Kivu is one of the provinces in the DRC with a higher fertility rate [26], it is more likely that the last morbid episode would be pregnancy or birth-related. A wide social acceptance and even social pressure to attend ANC or have a facility birth existing in the region may explain why women were more likely to use health centers.

### Strengths and limitations

To the best of our knowledge, this study is the first tracing recourse and motivation of choice in health care seeking in South Kivu, DRC. However, some authors focused their attention on the health-seeking behavior of sexual violence survivors [53] and the use of services starting from the health facilities’ side [54, 55]. We assessed the preference for different health structures to obtain an overview of patterns of resort and motivations for care-seeking to be able to meet the real needs of the population during a national health system’s reform process. However, the stigma associated with the use of traditional healers or prayer rooms may have induced a potential response bias or underreporting. The study did not determine the types of diseases that motivated the use of different healthcare structures, which could be an important topic for future research to inform sensitization and health care-seeking behavior change campaigns. Because of the limitations of the study design, we could not determine causality. Similarly, we did not seek the use of a tertiary structure or cost comparison while using the health facilities or alternative.

## Conclusion

The majority of the population used health centers or hospitals for their last illness episode. However, the proportion of those who patronized unrecognized care facilities is still considerable. Low cost and rapidity of services were the main motivations for those who used traditional healers and prayer rooms. Health system regulation is required to ensure the quality of all health services offered.

Community awareness campaigns focused on populations with lower education are still a key to encourage the use of integrated health facilities. To ensure more equity in primary health care access, a mapping and a re-distribution of all funders are necessary to provide quality care at minimal user fees. More research is also needed to better understand the gender dynamics and cultural aspects of health care seeking and patterns of the resort.

## Supplementary Information

**Additional file 1.** Questionnaire (English version).

## Data Availability

The generated dataset is not publicly available in order to preserve the confidentiality of information pertaining to identifiable individuals. However, it is stored and available from the corresponding author on reasonable request.

## Abbreviations

ANC: Antenatal care
BDOM: Bureau Diocésain des Œuvres Médicales
DHS: Demographic Health Survey
DRC: The Democratic Republic of the Congo
EPI: Expanded Program on Immunization
NGOs: Non-Governmental organization
RIPSEC: *Renforcement Institutionnel des Institutions pour les Politiques de santé basées sur l’évidence en République Démocratique du Congo*.

## References

[1] World HealthOrganization WHO, Organization. WH (2005). World health organization international health regulations.

[2] WHO (2019). Universal health coverage (UHC).

[3] Chol C, Negin J, Garcia-Basteiro A, Gebrehiwot TG, Debru B, Chimpolo M, Agho K, Cumming RG, Abimbola S (2018). Health system reforms in five sub-Saharan African countries that experienced major armed conflicts (wars) during 1990–2015: a literature review. Glob Health Action.

[4] Sayinzoga F, Bijlmakers L (2016). Drivers of improved health sector performance in Rwanda: a qualitative view from within. BMC Health Serv Res.

[5] Logie D, Rowson M, Ndagije F (2008). Innovations in Rwanda's health system: looking to the future. Lancet.

[6] Kapologwe NA, Meara JG, Kengia JT, Sonda Y, Gwajima D, Alidina S, Kalolo A (2020). Development and upgrading of public primary healthcare facilities with essential surgical services infrastructure: a strategy towards achieving universal health coverage in Tanzania. BMC Health Serv Res.

[7] Ngabo F, Nguimfack J, Nwaigwe F, Mugeni C, Muhoza D, Wilson DR (2012). Designing and implementing an innovative SMS-based alert system (RapidSMS-MCH) to monitor pregnancy and reduce maternal and child deaths in Rwanda. Pan Afr Med J.

[8] WHO. Health Systems in Africa Community: Perceptions and Perspectives. In: The Report of a Multi-Country Study. Brazzaville: WHO Regional Office for Africa; 2012.

[9] Cobos Muñoz D, Merino Amador P, Monzon Llamas L, Martinez Hernandez D, Santos Sancho JM (2017). Decentralization of health systems in low and middle income countries: a systematic review. Int J Public Health.

[10] Palagyi A, Marais BJ, Abimbola S, Topp SM, McBryde ES, Negin J (2019). Health system preparedness for emerging infectious diseases: a synthesis of the literature. Glob Public Health.

[11] Katung PY (2001). Socio-economic factors responsible for poor utilisation of the primary health care services in a rural community in Nigeria. Niger J Med.

[12] Musoke D, Boynton P, Butler C, Musoke MB (2014). Health seeking behaviour and challenges in utilising health facilities in Wakiso district, Uganda. Afr Health Sci.

[13] Shaikh BT, Hatcher J (2005). Health seeking behaviour and health service utilization in Pakistan: challenging the policy makers. J Public Health.

[14] Wiru K, Kumi-Kyereme A, Mahama EN, Amenga-Etego S, Owusu-Agyei S (2017). Utilization of community-based health planning and services compounds in the Kintampo north municipality: a cross-sectional descriptive correlational study. BMC Health Serv Res.

[15] Omeire E (2017). Factors affecting health seeking behaviour among rural dwellers in nigeria and its implication on rural livelihood. Eur J Soc Sci Stud.

[16] Uchendu OC, Ilesanmi OS, Olumide AE (2013). Factors influencing the choice of health care providing facility among workers in a local government secretariat in South-Western Nigeria. Ann Ibadan Postgrad Med.

[17] Magne C. Memoire Online - Etude des facteurs entravant la bonne fréquentation des structures sanitaires en milieu rural. Cas du CMA de Kongso Bafoussam III. 2012. https://www.memoireonline.com/09/13/7368/Etude-des-facteurs-entravant-la-bonne-frequentation-des-structures-sanitaires-enmilieu-rural-Cas.html. Accessed 12 July 2019.

[18] Tilahun H, Atnafu DD, Asrade G, Minyihun A, Alemu YM (2018). Factors for healthcare utilization and effect of mutual health insurance on healthcare utilization in rural communities of south Achefer Woreda, north west, Ethiopa. Health Econ Rev.

[19] Manzambi JK, Tellier V, Bertrand F, Albert A, Reginster JY, Balen H (2000). Les déterminants du comportement de recours au centre de santé en milieu urbain africain: résultats d'une enquête de ménage menée à Kinshasa, Congo. Trop Med Int Health.

[20] Marcellini A, Turpin J-P, Rolland Y, Ruffié S. Itinéraires thérapeutiques dans la société contemporaine. Corps et Cult. 2000;5. 10.4000/corpsetculture.710. Accessed 12 July 2019.

[21] James PB, Wardle J, Steel A, Adams J (2018). Traditional, complementary and alternative medicine use in sub-Saharan Africa: a systematic review. BMJ Glob Health.

[22] Stasse S, Vita D, Kimfuta J, da Silveira VC, Bossyns P, Criel B (2015). Improving financial access to health care in the Kisantu district in the Democratic Republic of Congo: acting upon complexity. Global Health Action.

[23] Ministère de la Santé RDC (2014). Rapport sur les Comptes Nationaux de la Santé.

[24] Ministère de la santé RDC (2006). Stratégie de Renforcement du système de santé (SRSS).

[25] Kalambayi NH, Van Leberghe W. Improving health system efficiency: Democratic Republic of Congo improving aid coordination in the health sector, vol. 2015. Geneva: WHO; 2015.

[26] MPSMRM, MSP, ICF (2014). Enquête Démographique et de Santé en République Démocratique du Congo 2013–2014.

[27] Ponsar F, Tayler-Smith K, Philips M, Gerard S, Van Herp M, Reid T (2011). No cash, no care: how user fees endanger health-lessons learnt regarding financial barriers to healthcare services in Burundi, Sierra Leone, Democratic Republic of Congo, Chad, Haiti, and Mali. Int Health.

[28] Laokri S, Soelaeman R, Hotchkiss DR (2018). Assessing out-of-pocket expenditures for primary health care: how responsive is the Democratic Republic of Congo health system to providing financial risk protection?. BMC Health Serv Res.

[29] Ministère de la santé RDC (2012). Normes et Directives relatives aux interventions à base communautaire pour la santé de la mère, du nouveau-né et de l’enfant.

[30] Ministère de la santé RDC. Normes sanitaires de la Zone de santé. Kinshasa: Ministère de la Santé RDC; 2002.

[31] Division Provincial de la santé Sud-Kivu (2017). Enumeration expanded Program on Immunization.

[32] Bureau Central de la Zone de Santé (BCZ) (2015). Plan de Développement de la Zone de Santé (PDSZ) de Walungu.

[33] Bureau Central de la Zone de Santé de Katana (2015). Plan de Développement de la Zone de Santé (PDSZ) de Katana.

[34] Hellen Keller International. Guide pour la réalisation des enquêtes de couverture post-événement de supplémentation en vitamine A, de déparasitage et de vaccination. Canada; 2014. Available on http://www.gava.org/content/user_files/2016/12/PECS_Manual_V2_-_3-14-14-French.pdf. Accessed 20 Apr 2017

[35] World Health Organization (2005). Immunization coverage cluster survey: reference manual.

[36] Robinson E, Crispino V, Ouabo A, Soung Iballa FB, Kremer R, Serbassi ME, van Lenthe M, Carrion Martin AI (2019). Mortality and health survey, Walikale, Democratic Republic of the Congo, 2017: an example of the use of survey data for humanitarian program planning. Confl Health.

[37] Schwartz D (1993). Méthodes statistiques à l’usage des médecins et des biologistes.

[38] World health organization (2015). Vaccination coverage cluster surveys: reference manual.

[39] Kuuire VZ, Bisung E, Rishworth A, Dixon J (2016). Health-seeking behaviour during times of illness: a study among adults in a resource poor setting in Ghana. J Public Health.

[40] White KL, Williams TF, Greenberg BG (1961). The ecology of medical care. N Engl J Med.

[41] Green LA, Fryer GE, Yawn BP, Lanier D, Dovey SM (2001). The ecology of medical care revisited. N Engl J Med.

[42] Thacker SB, Greene SB, Salber EJ (1977). Hospitalizations in a southern rural community: an application of the ‘ecology model’. Int J Epidemiol.

[43] Wambua JM, Mbayaki R, Munyao PM, Kabue MM, Mulindi R, Change PM, Ikamati R, Jahonga R, Ambalu R, Maranga W, Mudany M (2015). Client satisfaction determinants in four Kenyan slums. Int J Health Care Qual Assurance.

[44] Maini R, Van den Bergh R, van Griensven J, Tayler-Smith K, Ousley J, Carter D (2014). Picking up the bill - improving health-care utilisation in the Democratic Republic of Congo through user fee subsidisation: a before and after study. BMC Health Serv Res.

[45] Gyasi RM, Asante F, Segbefia AY, Abass K, Mensah CM, Siaw LP, Eshun G, Adjei POW (2015). Does spatial location matter? Traditional therapy utilisation among the general population in a Ghanaian rural and urban setting. Complement Ther Med.

[46] Gari A, Yarlagadda R, Wolde-Mariam M (2015). Knowledge, attitude, practice, and management of traditional medicine among people of burka Jato Kebele, West Ethiopa. J Pharm Bioallied Sci.

[47] Ministère de la santé RDC (2016). Plan national de développement sanitaire 2016–2020: vers une couverture universelle.

[48] Xu K, Evans DB, Kadama P, Nabyonga J, Ogwal PO, Nabukhonzo P, Aguilar AM (2006). Understanding the impact of eliminating user fees: utilization and catastrophic health expenditures in Uganda. Soc Sci Med.

[49] Ogunlesi TA, Olanrewaju DM (2010). Socio-demographic factors and appropriate health care-seeking behavior for childhood illnesses. J Trop Pediatr.

[50] Adongo W, Asaarik MJA (2018). Health seeking behaviors and utilization of healthcare services among rural dwellers in under-resourced communities in Ghana. Int J Caring Sci.

[51] Kim HB, Choi S, Kim B, Pop-Eleches C (2018). The role of education interventions in improving economic rationality. Science.

[52] Hahn RA, Truman BI (2015). Education improves public health and promotes health equity. Int J Health Serv.

[53] Bartels SA, Scott JA, Leaning J, Kelly JT, Joyce NR, Mukwege D, VanRooyen MJ (2012). Demographics and care-­seeking behaviors of sexual violence survivors in south Kivu Province, Democratic Republic of Congo. Disast Med Public Health Prepare.

[54] Casey SE, Mitchell KT, Amisi IM, Haliza MM, Aveledi B, Kalenga P, Austin J (2009). Use of facility assessment data to improve reproductive health service delivery in the Democratic Republic of the Congo. Confl Health.

[55] Soeters R, Peerenboom PB, Mushagalusa P, Kimanuka C (2011). Performance-based financing experiment improved health care in the Democratic Republic of Congo. Health Aff.

