# Supplementary material for: First recourse for care-seeking and associated factors among rural populations in the eastern Democratic Republic of the Congo
Source: BMC Public Health. 2021 Jul 10;21:1367. doi: 10.1186/s12889-021-11313-7 (PMC8272345; doi:10.1186/s12889-021-11313-7)
Supplement: Supplementary file 1 — Additional file 1. Questionnaire (English version). [file 12889_2021_11313_MOESM1_ESM.docx]

**Questionnaire (Community survey): First recourse for care-seeking and associated factors among rural populations in the eastern Democratic Republic of the Congo**

Wyvine Bapolisi Ansima^1^, Hermès Karemere ^1^, Freddy Ndogozi^4^, Aimé Cikomola ^1,2,4^, Ghislain Kasongo^5^, Albert Ntambwe^2,3^, Ghislain Bisimwa^1,2^

(1) Université Catholique de Bukavu, Faculté de Médecine, Ecole Régionale de Santé Publique de Bukavu, (2) Programme RIPSEC (Renforcement Institutionnel des Institutions pour les Politiques de santé basées sur l’évidence en République Démocratique du Congo). (3) Ecole de santé Publique de l’Université de Lubumbashi. (4) Division Provinciale de la santé du Sud Kivu. (5) Bureau central de la zone de santé de Walungu.

1. **IDENTITY OF THE PARTICIPANT**
2. Code of the participant :
3. Name ……………………………………………………Age (years)……………………………..
4. Date of birth____/____/_______
5. Sex M F
6. Katana Health Zone Walungu Health Zone
7. Matrimonial status : Single M married widow Divorced
8. Profession : Farmer Teacher State employee None
9. Household size Adults Number Less than 5yrs Number between 5 and17yrs
10. Position in family : a)responsible b)Dependent
11. Education level : none Primary Secondary Tertiary
12. Telephone number ………………………………..
13. Health zone………………………………. Village……………….
14. Date of the last consultation: ......./……/……… (month or at least year if the participants don't remember)

II. **SURVEY**

II.I.**ITINERARY**

1. In the last 15 days, were you sick? yes No
2. In the last 30 days, were you sick? yes No
3. In the last 30 days, was your partner or child or someone from your family sick? yes no
4. During your last illness episode, where did you seek for care?

a) Health center b) Hospital c) Private d) Traditional healer e) Prayer room

f) Self-medication g) I did not do anything

1. In case you went to hospital, why did you choose the hospital?

a) it was near my home b) I was looking for a specific specialist

c) I am more confident in a big hospital d) because of financial advantage I get there

e) a relative of mine is working there f) other to specify………………..

1. In case you went to the hospital, why did you choose to go to private?

a) it's cheaper b) the service is rapid c) because of financial advantage I get there

d) I was looking for a specific specialist e) Recommendation of a third party

f) other to specify…………………….

1. In case you went to traditional healer or prayer room, why did you choose to seek care from a traditional healer or in a prayer room?

a) it's cheaper b) the service is rapid c) I was looking for a specific specialist

d) Modern medicine couldn't heal the disease e) Recommendation of a third party

f) other to specify…………...

1. In case you used self medication, why did you self-medicate

a) it was cheaper b) it was easy c) everybody is doing it

d) a relative of mine or myself is a nurse/medical doctor

e) I don't trust health center If so, why don't you trust health center……………………………..

1. In the past 30 days, did you go to health centers seeking care? Yes No
2. Did you first go to the health center? Yes No
3. If not, where did you begin seeking for care at first instance?

a) self-medication b) traditional healer c) hospital d) private

e) If other, please specify:………..

II.2. **UTILIZATION OF HEALTH CENTER**

1. During your last episode of illness, did you use the health center when seeking care? Yes No
2. Were you hospitalized? Yes No
3. What services did you attend? a) Consultations b) Antenatal care c)Post-natal care

d) Pre-school e) Laboratory f) If other, please specify:……………...

1. If you have to estimate, how long (time in minutes) do you take to reach the health center?

a) less than 15 min b) between 15 min - 30 min c) between 30 min and 60 min

d) between 60 and 90 min e) between 90 - 120 min f) more than 120min

1. If you have to estimate, how often did you use the health center last month?

a) less than two times/month b) between 2 and 5 times/month c) more than 5 times/month d) never If never, why? ……………………..

II.3. **SATISFACTION OF SERVICES RECEIVED HEALTH CENTER**

1. If you have to estimate, how long (time in minutes) did you wait until the nurse received you at the health center? a) less than15 min b) between 15- 30 min c) between 30 - 60 min

d) more than 60 min e) I don't know

1. Did your wife/partner or a woman living in your household give birth recently (in the last year)?

Yes No

1. How many days the wife (woman) stayed at the health center after giving birth?

a) less than 3 days b) between 3 -7 days c) more than 7 days d) I don’t know

1. From your point of view, how do you judge the patients' fees charged by the health center?

a) very expensive b) expensive c) less expensive d) cheaper

1. according to the services provided at the health center, you can say that you were
2. a) totally satisfied b) satisfied c) unsatisfied d) totally unsatisfied Why?…………………………………………………..
3. According to you, how do you judge the cleanliness of the health centers?

a) very clean b) clean c) dirty d) very dirty

***Thank you for your time***
